# Prevalence and Associated Factors of Sleep Quality among Adults in Jimma Town, Southwest Ethiopia: A Community-Based Cross-Sectional Study

**DOI:** 10.1155/2018/8342328

**Published:** 2018-04-22

**Authors:** Hiwot Berhanu, Andualem Mossie, Samuel Tadesse, Daniel Geleta

**Affiliations:** ^1^Department of Medical Physiology, Jimma University, P.O. Box 378, Jimma, Ethiopia; ^2^Department of Child Health, John Snow International Research & Training Institute, P.O. Box 138998, Addis Ababa, Ethiopia

## Abstract

**Background:**

An estimated 150 million people worldwide and nearly 17% of the populations in the developing nations are currently suffering from sleep problems. The aim of the present study was to determine the prevalence and associated factors of sleep quality among adults in Ethiopia.

**Method:**

A cross-sectional study was conducted on 422 randomly selected adults using validated and pretested Pittsburgh Sleep Quality Index. Data were entered into EpiData and analyzed using SPSS version 20 considering bivariable (*P* value < 0.25) and multivariable (*P* < 0.05) logistic regression procedures at 95% confidence interval.

**Result:**

The overall prevalence of poor sleep quality (PSQI score > 5) was 65.4% with higher proportion among males (79 (63.0%)) and age group of 40–49 years (174 (28.6%)). A multivariable logistic regression analysis indicated that age category of 40–49 years (AOR = 2 [95% CI (1.1, 3.6)]) (*P* = 0.03), monthly income ≤ 1000 ETB (AOR = 2.2 [95% CI (14, 3.5)]) (*P* = 0.01), current khat chewing (AOR = 1.8 [95% CI (1.1, 3.1)]) (*P* = 0.03), daily khat chewing (AOR = 3.4 [95% CI (1.2, 11.1)]) (*P* = 0.04), and obesity (AOR = 1.2 [95% CI (1.3, 2.5)]) (*P* = 0.03) were identified as risk factors of poor sleep quality.

**Conclusion:**

The current study is informative for government to work on poverty reduction, create awareness for weight reduction, and develop legislation for khat control to prevent poor sleep quality.

## 1. Introduction

Sleep is part of what is called the sleep-wake cycle. This cycle, which consists of roughly 8 hours of nocturnal sleep and 16 hours of daytime wakefulness in humans, is controlled by a combination of two internal influences: sleep homeostasis and circadian rhythms [1]. In contrast to wakefulness, sleep is a period of inactivity and restoration of mental and physical function. It has been suggested that sleep provides time for entering information that has been acquired during periods of wakefulness into memory and for reestablishing communication between various parts of the brain. Sleep also is a time when other body systems restore their energy and repair their tissues [2] and is fundamental to wellbeing and optimal health [3–5]. People who get enough quality sleep have more energy, better cognitive function, healthier immune systems, and improved memory, alertness, attentiveness, and performance throughout the day [6]. Many hormones, such as growth hormone, are produced in a cyclic manner correlating with the sleep-wake cycle, suggesting that growth and tissue repair may occur during sleep. Another hormone produced towards the end of the night is the stress hormone cortisol, which begins to increase in preparation for the anticipated stress of the day, usually capped by a particularly large increase (up to 50%) about 20–30 minutes after waking, known as the cortisol awakening response [7]. Although sleep is one of the basic needs of human beings and is important to their health [8, 9], its problem has a wide range of causes including medical and psychological conditions. Some sleep problems are caused by restriction of the upper airway, while others are caused by genetic conditions. Other factors that affect sleep are age, medications, diet, and environmental factors, such as shift work. Sleep problem covers a broad spectrum of symptoms and is mostly characterized by one or more of symptoms like fatigue, inability to fall asleep at night, inability to stay asleep at night, excessive daytime sleepiness, loud snoring or gasping sounds during sleep, sleep attacks or unintended episodes of falling asleep, loss of muscle control or inability to move, and unusual behaviors such as sleep walking [10]. While sleep problems have existed for centuries, it is only within the last 3 to 4 decades that attention has focused on their diagnosis and classification [11]. To understand and assess sleep at different level, different methods have been developed to assess duration, distribution during the 24-hour day period, and quality of sleep [12]. Most often, the diagnosis of sleep problem usually is based on an adequate sleep history, physical examination, and a sleep diary or sleep log, where in some cases sleep laboratory studies may be needed to arrive at an accurate diagnosis [13]. Currently, the Pittsburgh Sleep Quality Index (PSQI) is an effective instrument used to measure the quality and patterns of sleep in adults by differentiating poor sleepers from good sleepers [14]. Sleep quality changes as a function of normal aging, in terms both of decreased duration and consolidation. Recent findings suggest that sleep quality plays a critical role in preserving cognitive function in older adults, reducing the risk of dementia [15], and it is identified to have bidirectional relationship with physical activity [4]. The National Sleep Foundation and consensus statement of the American Academy of Sleep Medicine and Sleep Research Society suggest that adults should sleep 7 or more hours per night on a regular basis to promote optimal health [5, 16, 17]. Not getting enough sleep can affect quality of life and untreated sleep problems can cause serious health problems and medical issues [18]. Researchers at the UK's University of Warwick Medical School conducted large-scale, multinational study of sleep disorders among eight countries in Asia and Africa which showed an overall rate of nearly 17% of the populations in these developing nations suffering from sleep problem. This is a figure not too far from the average 20% of the populations of the developed world that are believed to struggle with sleep problems of one form or another [19]. The levels of sleep difficulties experienced in developed nations like USA estimated that 50 to 70 million people are chronically suffering from sleep disorders [20]. The prevalence of sleep problems in mostly rural areas of developing nations is underestimated; researchers found a great deal of variation in the frequency of sleep difficulties. Some areas experienced very low levels of sleep disorders and others have conspicuous sleep problem. In South Africa, 31.3% of women and 27.2% of men reported difficulty with sleep. Overall rates of sleep problem in the remaining African nations of Tanzania, Ghana, and Kenya ranged between 8.3% and 12.7% and it is more prevalent than heart disease, cancer, AIDS, neurologic disease, breathing problems, diabetes, and gastrointestinal problems [19]. The prevalence of poor sleep quality (PSQI > 5) in general population as reported by study conducted among substance abusers in Cleveland, Ohio, was noted in 15% of the subjects [21]. Similarly, in China's population, the prevalence of poor sleep quality was reported to be 41.5% with a higher rate to be observed in elderly females (45.8%) than in elderly males (35.8%). The prevalence rate increased with age, from 32.1% (aged 60–69 years) to 52.5% (aged ≥ 80 years). The study also showed that less duration of education poses about 1.04 times risk and living alone was reported to cause 1.62 times poor sleep quality than their counterparts [22]. Another study conducted in China reported being female (45.8%) to be greater risk factor for poor sleep quality than being male (35.8%) [23, 24], whereas a study conducted in Lahore University showed the existence of similar level of poor sleep quality in both sexes [25]. A study done in Brazil indicates that individuals with higher socioeconomic status and education levels sleep better than those with lower socioeconomic status [23]. In Spain, the prevalence of poor sleep quality stands at 38.2%, where women were almost twice as likely as men to have poor quality, further indicating age to have direct association with a low quality of sleep [24]. Similarly, a cross-sectional study's result in Brazil revealed a positive correlation between percentage of slow-wave sleep and percentage of lean body mass in women. In men, awakenings during sleep were correlated positively with indices such as body mass index, waist circumference, hip circumference, and waist-to-hip ratio [23] and women were noted to sleep more than men [26]. Meanwhile, in other studies, women and younger age reported lesser sleep disturbance [27]. In a cross-sectional study done in China among 1023 nurses, the prevalence of poor sleep was 56.7%. Of these, 315 nurses (34.13%) were rotating-shift workers; multivariate logistic regression revealed that rotating shifts were independent risk factor for poor sleep quality [28, 29]. The prevalence of poor sleep quality was also reported in 54.7% of residents and 52.7% (51.8% among males and 56.9% among females) of college students who participated in a cross-sectional survey conducted to assess sleep quality in Thailand and Ethiopia, respectively [9, 30]. The previously conducted cross-sectional studies have identified that there is association between sex, residence, and substance use [31] (such as alcohol [32], khat [33–37], smoking [38, 39], caffeinated beverages [35, 40], and other stimulants) in different countries with poor sleep quality. Similarly, body composition [41], hypertension and Body mass index [42, 43], and diabetes mellitus [44] were reported by different studies of multiple countries to have association with poor sleep quality. Given the lack of merged studies on sleep quality in the previous review, in the current study, we performed assessment of sleep quality and associated factors. In addition, there are some limitations of previous studies to be highlighted. First, most of sleep studies were performed in Western countries, and few have been conducted in developing countries. So, due to differences in lifestyle and culture, findings obtained in Western countries might not be applicable to African populations in general and Ethiopia in particular. Second, while sleep quality contains quantity and quality parameters, the research on sleep is mostly focusing only on the quantity of sleep (sleep duration). Hence, this study is important to determine the problems and associated factors of sleep quality among adults population in the study area. Therefore, the aim of the present study was to determine the prevalence and associated factors of sleep quality among adults.

## 2. Methods

### 2.1. Study Area and Period

The study was conducted in Jimma town, which is one of the towns in Oromia national regional state, from 1 April to 28 April in 2016. Jimma is located 357 Km southwest of Addis Ababa. It is divided into 17 kebeles with a population of 120,960 and 32,192 households. A community-based cross-sectional study design was employed on randomly selected households. Sample size (*n*) was determined using single population proportion formula using 14.7% prevalence of poor sleep quality among households based on previous study at 95% confidence interval and 5% margin of error. Considering 2% to overcome design effect and 10% nonresponse rate, we have premeditated to register 422 participants for final sample size [19].

Simple random sampling using lottery method was used to select six study kebeles. After the study kebeles were identified, proportional sample was allocated to the selected kebeles using population proportional to size using lists of all households (from the existing lists of households from registration book of health extension workers) as a sampling frame. Hereby, the study participants were selected by systematic random sampling technique using computer-generated method from the sampling frame. Data were collected by 6 data collection trained nurses employed from the study area. Data collectors briefed objectives and purpose of the study to household heads and requested informed consent to collect data. After consent was obtained, face-to-face interview was conducted using a structured and pretested questionnaire for sociodemographic and other data. To evaluate sleep quality, participants were asked using Pittsburgh Sleep Quality Index (PSQI). PSQI differentiates “poor” from “good” sleep quality by measuring seven areas (components): subjective sleep quality, sleep latency, sleep duration, habitual sleep efficiency, sleep disturbances, use of sleeping medications, and daytime dysfunction over the last month [14]. The composite scores obtained indicate “good” (score < 5) and “poor” (score > 5) sleepers. A score of 0 to 3 is assigned to each of the seven scales (0 meaning no problem). The sum of the global PSQI scores ranged from 0 to 21. Finally, the subject whose global PSQI score was 5 or less was considered to have good sleep quality [9]. Finally, the data collectors measured weight (to the nearest 0.1 gm), height, hip and waist circumferences (to the nearest 0.1 centimeter), and BP (to the nearest 0.5 mmHg) of interviewed participants whose data were kept anonymous. Data were edited, coded, and entered into EpiData version 3.1 and exported to and analyzed by SPSS version 20. During the process of management, frequencies and percentages were calculated to describe the data by tables or figures. Bivariate analysis was performed separately using binary logistic regression to rank the relative importance of exposure variables with outcome variable using unadjusted odds ratios. The variables that have statistically significant (a *P* value of < 0.25) associations with the outcome variable in the bivariate analysis were further considered a candidate for stepwise multiple logistic regression model to control the effect of confounding variables. Finally, the variables that have statistically significant (a *P* value of < 0.05) associations with the sleep quality were considered as potential risk factors of sleep quality. The questionnaire was initially prepared in English, translated to local languages Afaan Oromo and Amharic, and retranslated to English by another person who was blind to the original questionnaire for consistency check. Pretesting of the questionnaire was made on 5% of sample size at one kebele (which was out of the main study kebeles) and corrective actions were taken accordingly by principal investigator. Once the quality of questionnaire was confirmed, two-day training was given for 6 data collectors and 1 supervisor by principal investigator focusing on the objective of the study and interview and measurement techniques. Completeness, accuracy, clarity, and consistency of every filled questionnaire were checked by the supervisor on daily basis. Checking for completeness and consistency of variables during data entry and analysis was continued before actual data analysis. Ethical clearance was obtained from Ethical Review Committee of Jimma University. The participants were informed and verbal consent was taken through signature or finger print.

## 3. Results

### 3.1. Sociodemographic Characteristics of Respondents

A total of 422 respondents were enrolled in the study, making 100% response rate. Of those enrolled, 257 (60.9%) were males, while 165 (39.1%) of them were females. The mean age of participants was 38.7 years (SD ± 12.5). More than half (69.4%) of participants were married. Majority of the study subjects were Oromo in ethnicity (68.5%) followed by Amhara (15.4%).

Among religion groups, 223 (52.8%) were Muslim, 28.2% Orthodox, and 16.1% other religions followers. Concerning educational status, 106 (25.1%) of respondents did not attend formal education, whereas 174 (41.2%) and 142 (33.6%) of them attended primary and secondary and above education, respectively. The of respondents (35.5%) were merchants. The average monthly income of the recruited households was 1786.10 ETB (with SD ± 1290.00) (Table 1).

### 3.2. Prevalence of Sleep Quality

The participants went to bed on average at 10:20 pm and rose in the morning at 5:50 am. Their average night sleep duration was 6.8 hours (SD ± 2.1). Seven components of sleep quality in the present study were assessed and identified their sleep status. The average sleep latency of the participants was 25 minutes (SD ± 15.0), and only 81 (19.2%) of the participants reported that their subjective sleep quality was very bad. In the present study, 329 (78.0%) of them reported that they had less than 7 hours of sleep per night, 217 (51.4%) had a low habitual sleep efficiency (<65%), and 100 (23.7%) used sleep medication within the past one month at the time of interview (Table 2). The mean scores of subjective sleep quality, sleep disturbances, and daytime dysfunction were 1.5 (SD ± 1.1), 1.4 (SD ± 0.9), and 0.9 (SD ± 0.8) hours, respectively. 276 (65.4%) participants were assessed as poor sleepers by global PSQI score greater than 5. The prevalence of poor sleepers was higher among males (174 (63.0%)) and the age group of 40–49 years (79 (28.6%)).

### 3.3. Factors Associated with Sleep Quality

#### 3.3.1. Sociodemographic Factors Associated with Sleep Quality

In the bivariable logistic regression analysis, variables such as age, sex, education, occupation, and household monthly income were associated with sleep quality (*P* < 0.25) as presented in Table 3; particularly, age categories of 40–49 [COR = 1.6, 95% CI (0.9, 2.8), *P* = 0.09] and ≥50 years [COR = 1.4, CI (0.9, 2.6), *P* = 0.23], being female [COR = 0.8, 95% CI (0.5, 1.2), *P* = 0.22], attaining primary education [COR = 1.4, 95% CI (0.9, 2.2), *P* = 0.14], being a merchant [COR = 1.1, 95% CI (0.3, 1.2), *P* = 0.17], being a housewife [COR = 1.1, 95% CI (0.3, 1.3), *P* = 0.22], and household monthly income less than 1000 ETB [COR = 2.2, 95% CI (1.5, 3.4), *P* = 0.22] were associated with poor sleep quality (Table 3).

### 3.4. Substances Use and Its Association with Sleep Quality

#### 3.4.1. Khat Chewing Practices

As per the current study result, 287 (68%) respondents chewed khat at least once in their life time. The overall past one month prevalence of khat chewing (current chewers) preceding the study was 51.2%. Of these, 100 (46.3%) chewers practiced khat chewing daily, whereas the remaining 57 (26.4%) practiced it 1–3 times per week and 59 (27.3%) practiced it 3–6 times per week. The amount of khat consumed at a time was estimated per cost in birr and 57 (26.4%) of the chewers consumed khat that costs >25 birr per ceremony (Table 4). The current study indicated that current khat chewers (54.0%) suffered from poor sleep quality [COR = 1.4, 95% CI (0.9, 2.3), *P* = 0.19]. Moreover, individuals who used to chew khat daily (46.6%) [COR = 1.7, 95% CI (0.8, 3.3), *P* = 0.16] and cost above 25 ETB (30.2%) [COR = 1.9, 95% CI (0.9, 3.9), *P* = 0.08] for khat have demonstrated higher proportion of poor sleep quality as indicated in Table 4.

#### 3.4.2. Alcohol Consumption

Higher proportions of current alcohol consumers (drink alcohol in the past 30 days preceding the study) (54%) were found to be poor sleepers, whereas association was observed in former consumers (12.8%) [COR = 0.6, 95% CI (0.3, 1.2), *P* = 0.12] and current consumers (38.4%) [COR = 1.1, 95% CI (0.7, 1.6), *P* = 0.08]. Beer was a kind of alcohol consumed by higher proportion (63.3%) of poor sleepers, whereas association was demonstrated by other kinds of alcohol (32.7%) [COR = 1.8, 95% CI (0.9, 2.7), *P* = 0.12] as shown in Table 4.

#### 3.4.3. Body Composition

Height, weight, and waist circumferences were taken and body mass index was calculated for every participant from these measurements. Higher proportion of poor sleep was identified among overweight (30.4%) groups. With regard to waist circumference, 70% of females and 40% of males were identified to be poor sleepers. Association between BMI and poor sleep quality was observed in obese group [7.6%, COR = 0.6, 95% CI (0.3, 13), *P* = 0.02] and waist circumference was observed in male sex [40%, COR = 3.0, 95% CI (1.3, 3.7), *P* = 0.08] as indicated in Table 5.

#### 3.4.4. Blood Pressure

Blood pressure of the subjects in a sitting position was taken from the left arm at their homes. For those with elevated BP, appointment was given on the next day and the second reading was taken. In this study, 20.7% of the study subjects with systolic hypertension (SBP ≥ 140 mmHg) and 13.8% with diastolic hypertension (DBP ≥ 90 mmHg) were identified to be poor sleepers. The result also showed association in both systolic [20.7%, COR = 1.3, 95% CI (0.8, 2.4), *P* = 0.22] and diastolic hypertension [13.8%, COR = 1.6, CI (0.8, 3.2), *P* = 0.12] (Table 5).

#### 3.4.5. Factors Independently Associated with Sleep Quality

All variables that had *P* value < 0.25 in the bivariate analysis were included in the multivariate analysis for backward logistic regression. From total variables included in the logistic regression model, four variables were found to be statistically significant at the level of *P* < 0.05. Accordingly, age, monthly income, BMI, and khat chewing status of study subject were demonstrated to have statistically significant association with sleep quality. Moreover, participants in the age category of 40–49 years were 2 times [AOR = 2, 95% CI (1.1, 3.6), *P* = 0.03] more likely to experience poor sleep quality than a person whose age was between 19 and 29 years. It was also identified that households with monthly income ≤1000 ETB were two times more likely to experience poor sleep quality than households with monthly income above 1000 ETB. Similarly, current khat chewers were about twice [AOR = 1.8, 95% CI (1.1, 3.1), *P* = 0.03] more likely to experience poor sleep quality than those who never chewed khat. Of the current khat chewers, subjects who chew daily were 3.4 times more likely to experience poor sleep quality [AOR = 3.4, 95% CI (1.2, 11.1), *P* = 0.04] than a person who used to chew khat 1–3 times per week. Finally, an obese person (BMI > 30 kg/cm^2^) is 1.5 times [AOR = 1.2, 95% CI (0.3, 2.5), *P* = 0.03] more likely to incur poor sleep quality than a person with normal range body mass index (BMI = 18.5–24.9 kg/cm^2^) as shown in Table 6. Some variables that demonstrated association in bivariable analysis like sex, educational status, occupation, alcohol consumption and kind of alcohol consumed, amount of coffee consumed per day, hypertension, and waist circumference have not demonstrated statistically significant association with sleep quality in final model. Therefore, they were not considered as potential independent factors of poor sleep quality.

## 4. Discussions

The current community-based study (used the validated PSQI) is the first to evaluate sleep quality and its associated factors among Jimma town's adult population. As a result, PSQI global score recognized 64.5% of participants to suffer poor sleep quality in preceding one month before the study. The proportion showed nearly similar prevalence and consistent result with the results of other studies in the literature [28]. However, the studies in Cleveland [21], china [22], Spain [24], Thailand [9], and Ethiopia (out of the current site) [30] reported lower proportion of sleep quality. Similarly, the overall rate of sleep quality in developing nations [19] including African nations (Tanzania, Ghana, and Kenya [20] in particular) reported lower proportion of the problem. To the level of our review, there was no study report higher than the current prevalence report. The reported variations between different studies may be influenced by different socioeconomic demands and cultural habits among the different population groups. Literature indicates that rural residents are more likely to report good levels of sleep quality when compared to urban residents [45] due to different factors such as sleep habits, sleep hygiene, cultural and racial differences, lifestyle, life quality, and stresses. The higher percentage of sleep problem in the current study could be explained to be the result of differences in the aforementioned factors and study setting (the current study included participants only from urban setting unlike studies conducted in America and China which included participants from both urban and rural settings).

The substantial higher proportion of the prevalence was observed among males (63.0%) unlike the study report conducted in South Africa [19] and China [22]. This might be due to the nature of the study participants in the area; males bear more responsibility to lead the family and substance users. In this study, sleep quality was significantly associated with age, monthly income, khat chewing, and body mass index in multivariable logistic regression analysis. This study identified that sleep quality is not continuously increasing with age as it was reported by the study conducted in China [22]. But age group of 40–49 years was identified to have higher risk for poor sleep quality than younger ages. This could be due to reduced melatonin levels as age increases compared with younger ages. Even though the clear mechanism is unknown, a variety of physiological and degenerative changes might cause degeneration of pathways from retina to pineal gland, and/or reduction of pinealocyte *β*-adrenergic receptor functions may contribute to lowering plasma melatonin levels and this may lead to poor sleep quality [37]. The other possible reason might be the testosterone decline at a rate of 1% per year leading to men being hypogonadal. In line with this fact, studies also indicated that lower levels of testosterone are connected to worse sleep consolidation in the form of reduced performance and increased frequency of awakenings [46]. Similarly, the possible reason might be the premenopausal/menopausal hormonal effects of estrogen in women. The possible mechanism could be that estrogen has been shown to decrease sleep latency, decrease the number of awakenings after sleep occurs, increase total sleep time, and decrease the number of cyclic spontaneous arousals. During the luteal (low estrogen) phase in premenopausal women, a twofold increase in the number of arousals occurs, particularly when both estrogen and progesterone levels are low [29].

Monthly income was another sociodemographic factor that showed relation with sleep quality. Individuals who earn ≤1000 ETB were twice poor sleeper than those with higher monthly income. A study conducted in Britain reported that people with higher socioeconomic status have a better quality of sleep than that of the poor [23]. This possibly occurs from mental satisfaction that could be related to higher quality of physical living environment including bedding and clothing which could affect thermoregulation. Current khat chewers have demonstrated association (1.8 times higher risk) with poor sleeping as of the current study's result. This result agrees with the studies conducted in Yemen and northwest Ethiopia [33, 34]. Furthermore, if a person chews khat every day, the quality of sleep deteriorates more. It was indicated that daily khat chewers had above three times risk of poor sleep quality than 1–3 times per week chewers. This study agrees with the study conducted in Ethiopia among college students [35]. This could be the result of psycho-stimulant and euphorigenic effects of khat impaired with sleep center [34]. Khat consumption leads to effects that are qualitatively similar to those of amphetamine, that is, increased blood pressure, a state of euphoria, and elation with feelings of increased alertness and arousal. This may be followed by depression, irritability, anorexia, and difficulty in sleeping. Frequent use of high doses may evoke psychotic reactions [47]. The euphoric effects of khat start after about one hour of chewing. Peak plasma levels of cathinone are obtained 1.5 to 3.5 hours after the onset of chewing. Cathinone is barely detectable in blood after eight hours. First-pass metabolism of cathinone in the liver leads to the formation of norephedrine which will worsen quality of sleep.

Finally, obesity was recognized to impair sleep quality. When compared to individual with normal BMI, obese individual carries 1.2 times more risk to exhibit poor sleep quality. This result agrees with the study conducted in USA [41] but contradicts the study conducted in Regensburg, Germany [48]. The variation of the results may occur from individuals' action to manage their situation (obesity). Some manage their diets (e.g., avoiding fat food in the evening) and practice frequent exercise to reduce putative negative influences on sleep quality, while others do not.

Data of this study were obtained through home-based face-to-face interviews by qualified nurses, reliable measurements, and cautious data handling. The study provided useful information that will inform policy makers to design a strategy to reduce the prevalence of poor sleep quality that affects health of the community at large. Although the study findings identified some important factors related to sleep quality, limitations should be noted. In line with this, study design may not allow for inferring cause and effect, as a PSQI questionnaire on irregular sleep history is prone to recall bias (not the gold standard to assess sleep quality). In summary, the proportion of poor sleep quality in the study's community seems to be high and commonly affects males. Further, age of 40–49 years, low income, khat chewing, and obesity were identified as risk factors of the problem. Therefore, it is indicative that government should work towards poverty reduction, create awareness for weight reduction, and develop legislation for khat control. Finally, as sleep habits may be a marker for health status and quality of life, longitudinal study would be needed to verify cause-effect relationship of sleep quality and presumed risk factors.

## Figures and Tables

**Table 1:** Sociodemographic characteristics of the sampled (*n* = 422) adults in Jimma town, southwest Ethiopia, 2016.

Variables	Number	%
Age		
19–29 years	120	28.4
30–39 years	114	27.0
40–49 years	112	26.5
50 years or above	76	18.0
Sex		
Male	257	60.9
Female	165	39.1
Religion		
Muslim	223	52.8
Orthodox	119	28.2
Others^*∗*^	80	19.0
Ethnic group		
Oromo	289	68.5
Amhara	68	16.1
Others^*∗∗*^	65	15.4
Educational status		
No formal education	106	25.1
Primary education	174	41.2
Secondary and above	142	33.6
Marital status		
Married	293	69.4
Unmarried	69	16.4
Others^*∗∗∗*^	60	14.2
Occupation		
Government employee	92	21.8
Housewife	127	30.1
Merchant	150	35.5
Others^*∗∗∗∗*^	53	12.6
Monthly income		
>1000 ETB	224	53.1
≤1000 ETB	198	46.9

^*∗*^Protestant, Catholic, Wakefata, and Jehovah. ^*∗∗*^Gurage, Tigre, Wolayta, Yem, and Dawro. ^*∗∗∗*^Divorced  and widowed. ^*∗∗∗∗*^Daily laborer, private employee, and home maid.

**Table 2:** Sleep quality and its components scores among adults in Jimma town, April 2016.

Variables (*n* = 422)	Value	Number	%
Sleep duration	>7 hours	93	22.0
	6-7 hours	72	17.1
	5-6 hours	57	13.5
	<5 hours	200	47.4
Sleep latency	0	65	15.4
	1	157	37.2
	2	112	26.5
	3	88	20.9
Daytime dysfunction	0	191	45.3
	1	138	32.7
	2	85	20.1
	3	8	1.9
Sleep efficiency	>85%	78	18.5
	75–84%	159	37.7
	65–75%	140	33.2
	<65%	45	10.7
Subjective sleep quality	Very good	93	22.0
	Fairly good	128	30.3
	Fairly bad	120	28.4
	very bad	81	19.2
Sleep disturbance	0	78	18.5
	1	159	37.7
	2	140	33.2
	3	45	10.7
Use of sleep medication	Not during the past month	375	88.9
	Less than once a week	30	7.1
	Once or twice a week	8	1.9
	Three or more times a week	9	2.1
Sleep quality score	Good sleep	146	34.6
	Poor sleep	276	65.4

**Table 3:** Association between sociodemographic factors and poor sleep quality among sampled adults of Jimma town, April 2016.

Variables	Global PSQI score			*P* value	COR (95% CI)
	Poor sleepers (*n* = 276)	Good sleepers (*n* = 146)	Total (*N*) = 422		
Age category					
19–29 years	72 (26.1)	48 (32.9)	120 (28.4)		1.0
30–39 years	73 (26.4)	41 (28.1)	114 (27.0)	0.53	1.2 (0.7, 2.1)
40–49 years	79 (28.6)	33 (22.6)	112 (26.5)	0.09^*∗*^	1.6 (0.9, 2.8)
≥50 years	52 (18.8)	24 (16.4)	76 (18.0)	0.23^*∗*^	1.4 (0.9, 2.6)
Sex					
Male	174 (63.0)	83 (56.8)	257 (60.9)		1.0
Female	102 (37.0)	63 (43.2)	165 (39.1)	0.22^*∗*^	0.8 (0.5, 1.2)
Education					
No formal	71 (25.7)	35 (24.0)	106 (25.1)	0.30	1.2 (0.8, 2.2)
Primary	119 (43.1)	55 (37.7)	174 (41.2)	0.14^*∗*^	1.4 (0.9, 2.2)
≥Secondary	86 (31.2)	56 (38.4)	142 (33.6)		1.0
Occupation					
Government employee	68 (24.6)	24 (16.4)	92 (21.8)		1.0
Housewife	86 (31.2)	41 (28.1)	127 (30.1)	0.22^*∗*^	0.6 (0.3, 1.3)
Merchant	88 (31.9)	62 (42.5)	150 (35.5)	0.17^*∗*^	0.5 (0.3, 1.2)
Others^*∗*^	34 (12.3)	19 (13)	53 (12.6)	0.32	0.7 (0.4, 1.5)
Monthly income					
>1000 ETB	128 (46.4)	96 (65.8)	224 (53.1)		1.0
≤1000 ETB	148 (53.6)	50 (34.2)	198 (46.9)	0.00^*∗*^	2.2 (1.5, 3.4)

^*∗*^ *P* value < 0.25.

**Table 4:** Substances use and its association with sleep quality among adults in Jimma town, southwest Ethiopia, April 2016.

Variables	Global PSQI score							
	Poor sleepers (*n* = 276)		Good sleepers (*n* = 146)		Total (*N*) 422		*P* value	COR (95% CI)
	*n*	%	*n*	%	*n*	%		
Khat chewing status								
Never	84	30.4	51	34.9	135	32.0		1.0
Former	43	15.6	28	19.2	71	16.8	0.81	0.9 (0.5, 1.7)
Current	149	54.0	67	45.9	216	51.2	0.19^*∗*^	1.4 (0.9, 2.3)
Frequency of khat chewing								
1–3 times/wk	36	24.2	21	31.3	57	26.4		1.0
3–6 times/wk	39	26.2	20	29.9	59	27.3	0.74	1.1 (0.4, 2.4)
Daily	74	49.6	26	38.8	100	46.3	0.16^*∗*^	1.7 (0.8, 3.3)
Cost of khat per ceremony								
≤15 Birr	86	57.7	44	65.7	130	60.2		1.0
16–25	18	12.1	11	16.4	29	13.4	0.67	0.8 (0.3, 1.9)
>25	45	30.2	12	17.9	57	26.4	0.08^*∗*^	1.9 (0.9, 3.9)
Alcohol consumption status								
Never	137	49.6	69	47.3	206	48.8		1.0
Former	30	10.9	24	16.4	54	12.8	0.12^*∗*^	0.6 (0.3, 1.2)
Current	109	39.5	53	36.3	162	38.4	0.08^*∗*^	1.1 (0.7, 1.6)
Kind of alcohol consumed								
Beer	69	63.3	40	75.5	109	67.3		
Others^*∗*^	40	36.7	13	24.5	53	32.7	0.12^*∗*^	1.8 (0.9, 3.7)
Amount of coffee consumed per day								
1-2 cups/day	108	41.9	57	42.5	165	42.1		1.0
3-4 cups/day	83	32.2	54	40.3	137	34.9	0.38	0.8 (0.5, 1.2)
5 ≥ cups/ day	67	26.0	23	17.2	90	23.0	0.14^*∗*^	1.5 (0.9, 2.7)

^*∗*^ *P* value < 0.25; others^*∗*^ represent Tela, Tej, and Katicala.

**Table 5:** Body composition and BP measurements and their association with sleep quality among sampled adults in Jimma town, April 2016.

Variables	Global PSQI score						*P* value	COR (95% CI)
	Poor sleepers (*n* = 276)		Good sleepers (*n* = 146)		Total (422)			
	*n*	%	*n*	%	*n*	%		
Categories of BMI (kg/cm^2^)								
Normal	123	44.6	60	41.1	183	43.4		1.0
Underweight	48	17.4	20	13.7	68	16.1	0.61	1.2 (0.6, 2.1)
Overweight	84	30.4	50	34.2	134	31.8	0.40	0.8 (0.5,1.4)
Obese	21	7.6	16	11.0	37	8.8	0.02^*∗*^	0.6 (0.3, 13)
Male waist circumference								
<90 cm	112	60.0	23	33.0	135	53.0		1.0
≥90 cm	74	40.0	46	67.0	120	47.0	0.08^*∗*^	3.0 (1.3, 3.7)
Female waist circumference								
<80 cm	30	30.0	26	40.0	56	33.5		1.0
≥80 cm	72	70.0	39	60.0	111	66.5	0.25	0.6 (0.8, 3.2)
SBP								
Normal	219	79.3	123	84.2	342	81.0		1.0
Hypertension	57	20.7	23	15.8	80	19.0	0.22^*∗*^	1.3 (0.8, 2.4)
DBP								
Normal	238	86.2	133	91.1	371	87.9		1.0
Hypertensive	38	13.8	13	8.9	51	12.1	0.12^*∗*^	1.6 (0.8, 3.2)

^*∗*^ *P* value < 0.25.

**Table 6:** Independent factors associated with poor sleep quality (sampled = 422) among adults in Jimma town, April 2016.

Variables	Global PSQI score			Bivariable result		Multivariable result	
	Poor sleepers (*n* = 276)	Good sleepers (*n* = 146)	Total	*P* value	COR (95% CI)	*P* value	AOR (95% CI)
	*n* (%)	*n* (%)	*n* (%)				
Age category							
19–29 years	72 (26.1)	48 (32.9)	120 (28.4)		1.0		1.0
30–39 years	73 (26.4)	41 (28.1)	114 (27.0)	0.53	1.2 (0.7, 2.1)	0.83	0.9 (0.5,1.7)
40–49 years	79 (28.6)	33 (22.6)	112 (26.5)	0.09	1.6 (0.9, 2.8)	0.03^*∗∗*^	2.0 (1.1, 3.6)
50–64 years	52 (18.8)	24 (16.4)	76 (18.0)	0.03	1.4 (0.9, 2.6)	0.37	1.4 (0.7, 2.8)
Monthly income							
>1000 ETB	128 (46.4)	96 (65.8)	224 (53.1)		1.0		1.0
≤1000 ETB	148 (53.6)	50 (34.2)	198 (46.9)	0.00	2.2 (1.5, 3.4)	0.01^*∗∗*^	2.2 (1.4, 3.5)
Khat chewing status							
Never	84 (30.4)	51 (34.9)	135 (32.0)		1.0		1.0
Former	43 (15.6)	28 (19.2)	71 (16.8)	0.81	0.9 (0.5, 1.7)	0.33	1.4 (0.7, 2.7)
Current	149 (54.0)	67 (45.9)	216 (51.2)	0.19	1.4 (0.9, 2.3)	0.03^*∗∗*^	1.8 (1.1, 3.1)
Frequency of khat chewing							
1–3 times/wk	36 (24.2)	21 (31.3)	57 (26.4)		1.0		1.0
3–7 times/wk	39 (26.2)	20 (29.9)	59 (27.3)	0.38	0.8 (0.5, 1.2)	0.18	2.3 (0.7, 7.8)
Daily	74 (49.6)	26 (38.8)	100 (46.3)	0.14	1.5 (0.9, 2.7)	0.04^*∗∗*^	3.4 (1.2, 11.1)
Categories of BMI (kg/cm^2^)							
Normal	123 (44.6)	60 (41.1)	183 (43.4)		1.0		
Underweight	48 (17.4)	20 (13.7)	68 (16.1)	0.61	1.2 (0.6, 2.1)	0.05	0.9 (0.3, 2.2)
Overweight	84 (30.4)	50 (34.2)	134 (31.8)	0.40	0.8 (0.5,1.4)	0.06	1.1 (0.6, 1.8)
Obese	21 (7.6)	16 (11.0)	37 (8.8)	0.02^*∗*^	0.6 (0.3, 13)	0.03^*∗∗*^	1.2 (1.3, 2.5)

^*∗*^ *P* value < 0.25; ^*∗∗*^statistically significant at *P* value <0.05.
